# Cross-sectional analysis characterizing the use of rank preserving structural failure time in oncology studies: changes to hazard ratio and frequency of inappropriate use

**DOI:** 10.1186/s13063-023-07412-y

**Published:** 2023-06-03

**Authors:** Vinay Prasad, Myung Sun Kim, Alyson Haslam

**Affiliations:** 1 Department of Epidemiology and Biostatistics, University of California San Francisco, 550 16th St, 2nd Fl, San Francisco, CA 94158 USA; 2 Oregon Health and Science University, 3181 SW Sam Jackson Park Road, Portland, OR 97239 USA

**Keywords:** RPSFT, Overall survival, Hazard ratio

## Abstract

**Background:**

Rank preserving structural failure time (RPSFT) is a statistical method to correct or adjust for crossover in clinical trials, by estimating the counterfactual effect on overall survival (OS) when control arm patients do not receive the interventional drug when their tumor progresses. We sought to examine the strength of correlation between differences in uncorrected and corrected OS hazard ratios and percentage of crossover, and characterize instances of fundamental and sequential efficacy.

**Methods:**

In a cross-sectional analysis (2003–2023), we reviewed oncology randomized trials that used RPSFT analysis to adjust the OS hazard ratio for patients who crossed over to an anti-cancer drug. We calculated the percentage of RPSFT studies evaluating a drug for fundamental efficacy (with or without a standard of care (SOC)) or sequential efficacy and the correlation between the OS hazard ratio difference (unadjusted and adjusted) and the percentage of crossover.

**Results:**

Among 65 studies, the median difference between the uncorrected and corrected OS hazard ratio was −0.1 (quartile 1, quartile 3 : −0.3 to −0.06). The median percentage of crossover was 56% (quartile 1, quartile 3: 37% to 72%). All studies were funded by the industry or had authors who were employees of the industry. Twelve studies (19%) tested a drug’s fundamental efficacy when there was no SOC; 34 studies (52%) tested a drug’s fundamental efficacy when there was already a SOC; and 19 studies (29%) tested a drug’s sequential efficacy. The correlation between the uncorrected and corrected OS hazard ratio difference and the percentage of crossover was 0.44 (95% CI: 0.21 to 0.63).

**Conclusions:**

RPSFT is a common tactic used by the industry to reinterpret trial results. Nineteen percent of RPSFT use is appropriate. We recognize that while crossover can bias OS results, the allowance and handling of crossover in trials should be limited to appropriate circumstances.

**Supplementary Information:**

The online version contains supplementary material available at 10.1186/s13063-023-07412-y.

## Background

Crossover, when a patient in the control arm receives the interventional drug upon tumor progression, can introduce bias in oncology trials, especially since trial results are often interpreted with the intention-to-treat principle, where data are analyzed based on treatment assignment and not actual treatment receipt. This can lead to an underestimation of the drug’s effect if a drug truly reduces mortality. Rank preserving structural failure time (RPSFT) and inverse probability of censoring weighting are two popular statistical methods [1, 2] to correct or adjust for crossover in clinical trials, by estimating the counterfactual effect on overall survival (OS) when control arm patients do not receive the interventional drug when their tumor progresses.

In short, an acceleration factor is applied to a counterfactual event time, namely the duration of time an individual receives the interventional treatment. The acceleration factor is identified through a grid search (G-estimation) procedure and approximates the decrease in an individual’s survival time if the control treatment had been used instead of the interventional treatment [3].

However, the RPSFT correction makes some assumptions, namely the common treatment effect assumption (i.e., the treatment effect is equal for all patients regardless of when they receive treatment) and the randomization assumption (i.e., all patients have the same opportunity to receive treatment) [4]. Not meeting these assumptions can result in an ineffective RPSFT analysis. Along with these two assumptions, there is an underlying assumption that when crossover occurs, the drug used at tumor progression has demonstrated OS benefit for the given indication. In other words, is it appropriate to cross the patient over to the drug being tested? We have previously described situations for when this is appropriate and when it is not [5]. Crossover is desirable when an experimental drug has already proven beneficial in a latter line of therapy or is standard of care in the latter line. In this situation, the patient receives an established standard of care. Conversely, crossover is problematic when the fundamental efficacy of the experimental agent has not been established in any prior study, thus patients may receive inferior treatment.

In this present study, we sought to review published RPSFT analyses in oncology drug trials, to characterize when this type of analysis is being done (fundamental or sequential efficacy), to examine the strength of correlation between differences in uncorrected and corrected OS hazard ratios and percentage of people who crossover, and to determine whether RPSFT contributes to notable difference in OS significance between the uncorrected and corrected analyses.

## Methods

### Article search

We searched Embase, PubMed, and Google Scholar for studies that used RPSFT to adjust for OS due to crossover. For Embase and PubMed, we used the search terms: “rank preserving structural failure time” OR (rank AND preserving AND structural AND (“failure”/exp OR failure) AND (“time”/exp OR time)). For Google Scholar, we used the search terms: “rank preserving structural failure time” AND (cancer OR oncology). We searched for studies published since 2003. The searches were made on April 10, 2023.

Included studies needed to (1) use RPSFT to adjust for OS due to crossover in the study’s analysis; (2) include patients with cancer; (3) be an analysis of a randomized trial; (4) be written in English; and (5) have an intervention with an anti-tumor drug. Excluded studies (1) used RPSFT to adjust for another outcome besides OS; (2) were economic studies that did not report an adjusted HR; (3) were simulation or statistical methodology studies; (4) were a review article or summary of a prior RPSFT analysis; (5) used a method to adjust for crossover that was not RPSFT; (6) compared RPSFT-adjusted OS between two different trials; or (7) were an adjustment on a non-drug intervention. Articles could be in the form of abstracts if they met the inclusion/exclusion criteria. Initially, we allowed multiple reports on the same trial, as long as the reports were published or presented in separate analyses (e.g., different years, titles, and journal/conference). This allowed us to see if some trials had more RPSFT analyses than others. For the main analysis, we restricted the data so that each trial was a single observation.

### Data abstraction

We abstracted data on the year of study publication, tumor type, intervention and control agents, percent crossover, uncorrected and corrected median OS for both the intervention and control arms, the uncorrected and corrected hazard ratios, the trial registry number, the study funder, median age, percent of male participants, number of patients randomized to each arm, blinding status (open vs. patient blinding), and median follow-up. We further abstracted data on dates of enrollment, whether crossover was permitted, and if an RPSFT analysis was planned *a priori*. If these data were not reported in the RPSFT study, we looked in the original study report and protocol (using the trial registry number) to see if these data were reported there.

We then searched to see if the drug was US Food and Drug Administration (FDA) approved for the indication tested in the study and the year of approval. We classified drugs as being tested for fundamental efficacy or sequential efficacy, based on the following criteria. In instances of fundamental efficacy, the drug was not on the market at time of study start date or had not been shown to have efficacy in latter lines of the same tumor type. Fundamental efficacy was then further categorized as situations where there was an established/existing standard of care (i.e., a drug being tested in second line treatment when other second-line treatments have already been approved for the tumor type) or situations where no standard of care existed (e.g., GIST [gastrointestinal stromal tumor] pre-imatinib approval). Sequential efficacy was defined as a drug that had already been tested and approved in a latter line but was being tested in an earlier line or upfront use (i.e., the drug was approved for second line treatment, but being tested for first line). Classification of fundamental or sequential testing was determined by two separate reviewers (AH and MSK).

### Statistical analysis

We calculated frequencies (percentages) and medians (quartile 1, quartile 3 [Q1, Q3] for the characteristics of the studies. We used chi-square and Kruskal–Wallis tests, for categorical and continuous variables, to determine statistical significance between fundamental (with or without standard of care) and sequential efficacy categories. We used Pearson’s correlation to determine the association between the percentage of participants who crossed over and the difference between the uncorrected and corrected OS hazard ratio and plotted the values. We calculated an unadjusted linear regression line to determine the slope of the correlation, as well as an adjusted line, adjusted for the total number of participants, the randomization ratio (1:1, 2:1, etc.), blinded vs open status of drug receipt, and fundamental vs sequential efficacy status. For the regression models, the change in OS hazard ratio was the dependent variable and the percentage of participants who crossed over was the independent variable. We checked model assumptions with four tests: residuals vs fitted for linearity; normal Q-Q plot for normality; scale-location for homogeneity; and residuals vs leverage for influential cases (Supplemental Figure 1). For interpreting the correlation coefficients, we defined high correlation as *R* ≥ 0.85, low correlation as *R* ≤ 0.7, and results of *R* < 0.85 and > 0.7 were considered moderate or unclear correlation [6].

We also ran a Fleiss’s kappa test to determine the agreement between uncorrected and corrected OS hazard ratios being significant or not. Values between 0.01–0.20 indicated slight agreement; 0.21–0.40 indicated fair agreement; 0.41–0.60 indicated moderate agreement; 0.61–0.80 indicated substantial agreement; and 0.81–0.99 indicated almost perfect agreement [7, 8]. We also calculated an intraclass correlation coefficient (ICC) to determine the correlation between the uncorrected and corrected hazard ratio and displayed the agreement in a Bland-Altman plot. We used R statistical software (version 4.2.1) [9] for these analyses, package ‘irr’ for the kappa and ICC statistic and package ‘ggplot’ for the Bland-Altman plot [10, 11]. To show publication bias in both uncorrected and corrected hazard ratio estimates, we used the ‘meta’ package to develop contour-enhanced funnel plot [12]. We used an alpha level of 0.05 for determining statistical significance.

In accordance with 45 CFR §46.102(f), this study was not submitted for institutional review board approval because it involved publicly available data and did not involve individual patient data.

## Results

Our search resulted in 160 Embase articles, 45 PubMed articles, and 801 Google Scholar articles (Supplemental Figure 2). After excluding exact duplicate searches (i.e., matching titles) and articles not meeting our inclusion criteria, we found 111 articles and abstracts meeting our criteria. Of the 111 articles, there were 46 articles that were duplicate RPSFT analyses but were presented in different years or journals/conferences, resulting in 65 unique RPSFT analyses. Most trials had a single publication on RPSFT analysis (median = 1; mean = 1.8) but had as many as 5 publications/presentations of the same trial.

For the 65 unique studies (Table 1), there was a median of 361 participants (Q1, Q3: 233 to 512). The median age was 61 years (Q1, Q3: 58 years to 64 years)

**Table 1:** Characteristics of oncology drug studies reporting rank preserving structural failure time analysis

	**All studies (*N*=65)**	**Fundamental, no SOC** **(*N*=12)**	**Fundamental with SOC** **(*N*=34)**	**Sequential** **(*N*=19)**	***p*-value**^**1**^
**Tumor type, *n* (%)**					<0.001
**Breast**	7 (10.8)	0	7 (20.6)	0	
**Colorectal**	3 (4.6)	0	0	3 (15.8)	
**GIST**	4 (6.2)	4 (33.3)	0	0	
**CLL/SLL**	4 (6.2)	0	4 (11.8)	0	
**Melanoma**	5 (7.7)	1 (8.3)	4 (11.8)	0	
**Myelofibrosis**	2 (3.1)	2 (16.7)	0	0	
**Multiple myeloma**	5 (7.7)	0	3 (8.8)	2 (10.5)	
**Neuroendocrine**	3 (4.6)	3 (25.0)	0	0	
**Non-small cell lung cancer**	13 (20.0)	0	6 (17.6)	7 (36.8)	
**Ovarian**	2 (3.1)	0	0	2 (10.5)	
**Prostate**	5 (7.7)	0	3 (8.8)	2 (10.5)	
**Renal cell carcinoma**	4 (6.2)	1 (8.3)	2 (6.1)	1 (5.3)	
**Thyroid**	3 (4.6)	1 (8.3)	2 (5.9)	0	
**Other**	5 (7.7)	0	3 (8.8)	2 (10.5)	
**Randomization ratio,* n* (%)**					0.18
**1:1**	37 (56.9)	5 (41.7)	17 (50.0)	15 (78.9)	
**2:1**	27 (41.5)	7 (58.3)	16 (47.1)	4 (21.1)	
**3:1**	1 (1.5)	0	1 (2.9)	0	
**Open label, *n* (%)**	29 (44.6)	2 (16.7)	17 (50.0)	10 (52.6)	0.08
**FDA approved for indication (yes),* n* (%)**	54 (83.1)	11 (91.7)	26 (76.5)	17 (98.5)	0.33
**Medical writers, *n* (%)**					0.33
**Yes**	44 (67.7)	7 (58.3)	26 (76.5)	11 (57.9)	
**No**	3 (4.6)	0	1 (2.9)	2 (10.5)	
**Not indicated**	18 (27.7)	5 (41.7)	7 (20.6)	6 (31.6)	
**Author as employee of company (yes), *n* (%)**	58 (89.2)	10 (82.3)	31 (91.2)	17 (89.5)	0.26
**Age of participants, median (Q1, Q3)**	61 (58 to 64)	62 (58 to 64)	62 (55 to 64)	60 (58 to 62)	0.82
**Total number of participants, median (Q1, Q3)**	361 (233 to 512)	311 (214 to 373)	392 (259 to 519)	359 (242 to 690)	0.20
**Difference in adjusted and unadjusted overall survival hazard ratio, median (Q1, Q3)**	-0.1 (-0.3 to -0.6)	-0.3 (-0.3 to -0.02)	-0.1 (-0.2 to -0.3)	-0.09 (-0.2 to -0.4)	0.02
**% Trials with numerically higher unadjusted overall survival hazard ratio, *n* (%)**	60 (92.3)	12 (100)	31 (91.2)	17 (89.5)	0.39
**% Crossover, median (Q1, Q3)**	55.7 (36.5 to 71.6)	75.3 (59.3 to 84.2)	53.0 (38.0 to 67.4)	46.6 (33.2 to 67.4)	0.02
**Median overall survival for intervention arm, median (Q1, Q3)**	23.9 (17.8 to 36.8)	18.4 (17.6 to 33.6)	24.7 (16.2 to 42.6)	24.0 (22.9 to 32.8)	0.74
**Median overall survival for control arm (uncorrected), median (Q1, Q3)**	20.5 (14.5 to 36.3)	17.4 (16.2 to 36.3)	19.4 (12.0 to 22.3)	30.3 (21.5 to 36.7)	0.11
**Median overall survival for control arm (corrected), median (Q1, Q3)**	15.9 (9.8 to 27.4)	10.9 (9.6 to 17.6)	12.3 (7.8 to 18.3)	26.6 (17.1 to 35.1)	0.04
**Change in significance of overall survival hazard ratio (corrected vs. uncorrected), yes *n* (%)**	41 (64.1)	3 (25.0)	22 (66.7)	16 (84.2)	0.003
**RPSFT analysis stipulated in protocol**					0.36
**Yes**	22 (33.8)	2 (16.7)	13 (38.2)	7 (36.8)	
**No**	29 (44.6)	5 (41.7)	16 (47.1)	8 (42.1)	
**No protocol available**	14 (21.5)	5 (41.7)	5 (14.7)	4 (21.1)	
**Year of study publication, *n* (%)**					0.07
**2008**	1 (1.5)	1 (8.3)	0	0	
**2011**	1 (1.5)	0	0	1 (5.3)	
**2012**	3 (4.6)	2 (16.7)	1 (2.9)	0	
**2013**	2 (3.1)	0	2 (5.9)	0	
**2015**	4 (6.2)	1 (8.3)	3 (8.8)	0	
**2016**	11 (16.9)	4 (33.3)	4 (11.8)	3 (15.8)	
**2017**	6 (9.2)	2 (16.7)	0	4 (21.1)	
**2018**	5 (7.7)	0	4 (11.8)	1 (5.3)	
**2019**	5 (7.7)	0	3 (8.8)	2 (10.5)	
**2020**	7 (10.8)	0	5 (14.7)	2 (10.5)	
2021	11 (16.9)	2 (16.7)	7 (20.6)	2 (10.5)	
**2022**	9 (13.8)	0	5 (14.7)	4 (21.1.)	

*GIST* Gastrointestinal stromal tumors, *CLL* Chronic lymphocytic leukemia, *SLL* Small lymphocytic lymphoma, *SOC* Standard of care, *Q1, Q3* Quartile 1, quartile 3, *RPSFT* Rank preserving structural failure time

^1^*P*-values are derived from chi-square and Kruskal–Wallis tests, for categorical and continuous variables, respectively

The most common tumor types studied were non-small cell lung cancer (*n*=13; 20%), breast (*n*=7; 11%), and myeloma (*n*=5; 8%). Thirty-seven studies (57%) had a 1:1 randomization ratio, 27 studies (42%) had a 2:1 ratio, and 1 (2%) had a 3:1 ratio. Twenty-nine (45%) studies were open-label studies.

The median difference between the uncorrected and corrected OS hazard ratio was −0.1 (Q1, Q3: −0.3 to −0.6). In other words, the hazard ratio became more favorable by 0.1, after adjustment. The median percentage of participants who crossed over was 56% (Q1, Q3: 37% to 72%). All 65 studies were funded by industry (53/53 studies reporting funding source) or had authors (94%; *n*=61) who were employees of the company that manufactured the study drug.

Sixty-eight percent of studies used medical writers (90% of full-length articles), but 28% did not include acknowledgements or a section on who wrote the article (e.g., abstracts only).

Twelve studies (19%) tested a drug’s fundamental efficacy when there was no standard of care; 34 studies (52%) tested a drug’s fundamental efficacy when there was already a standard of care; and 19 studies (29%) tested a drug that was already used in a latter line, being moved upfront, where some percentage of the control arm eventually received that therapy (sequential testing).

**Fig. 1:**
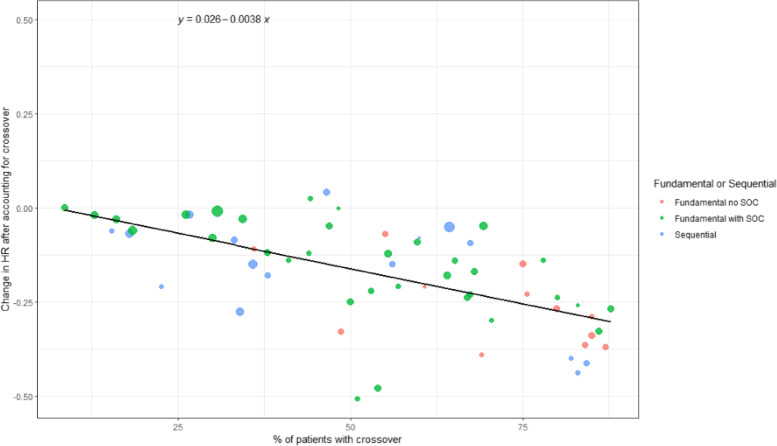
Correlation between the difference in uncorrected and corrected OS hazard ratios and % patients with crossover in rank preserving structural failure time analyses. The size of the circle is weighted by the sample size. One observation was removed due to undue influence

After removing one outlier, the correlation between the uncorrected and corrected OS hazard ratio and the percentage of individuals who crossed over was 0.62 (95% CI: 0.42 to 0.76; *R*^2^=0.38; *p*<0.001; Fig. 1). When adjusting for the number of participants, randomization ratio, blinding status, and fundamental or sequential efficacy, the correlation was similar (*r*=0.66; 95% CI: 0.48 to 0.79; *R*^2^: 0.43; *p*=0.0001). None of the other variables were significantly associated with the difference in hazard ratio. Without removing the outlier, the unadjusted correlation was 0.44 (95% CI: 0.21 to 0.63; *R*^2^: 0.19; *p*=0.0004).

The median uncorrected and corrected hazard ratio difference among trials testing fundamental efficacy without a standard of care was −0.3, among trials testing fundamental efficacy with standard of care, it was −0.1, and among trials testing a drug in sequential order, it was −0.09.

**Fig. 2:**
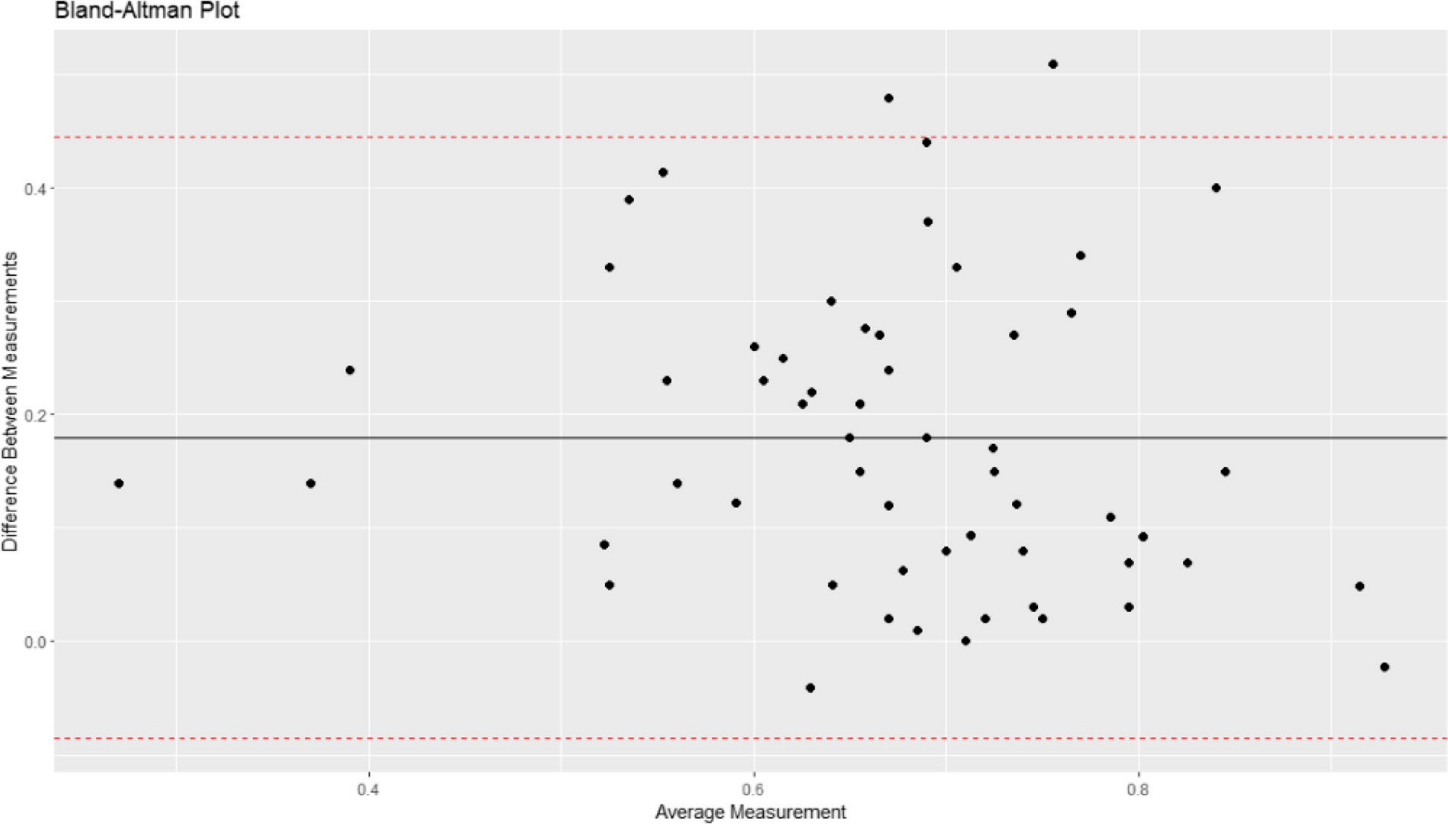
Bland-Altman plot showing agreement between the uncorrected and corrected hazard ratios in oncology rank preserving structural failure time analyses

**Fig. 3:**
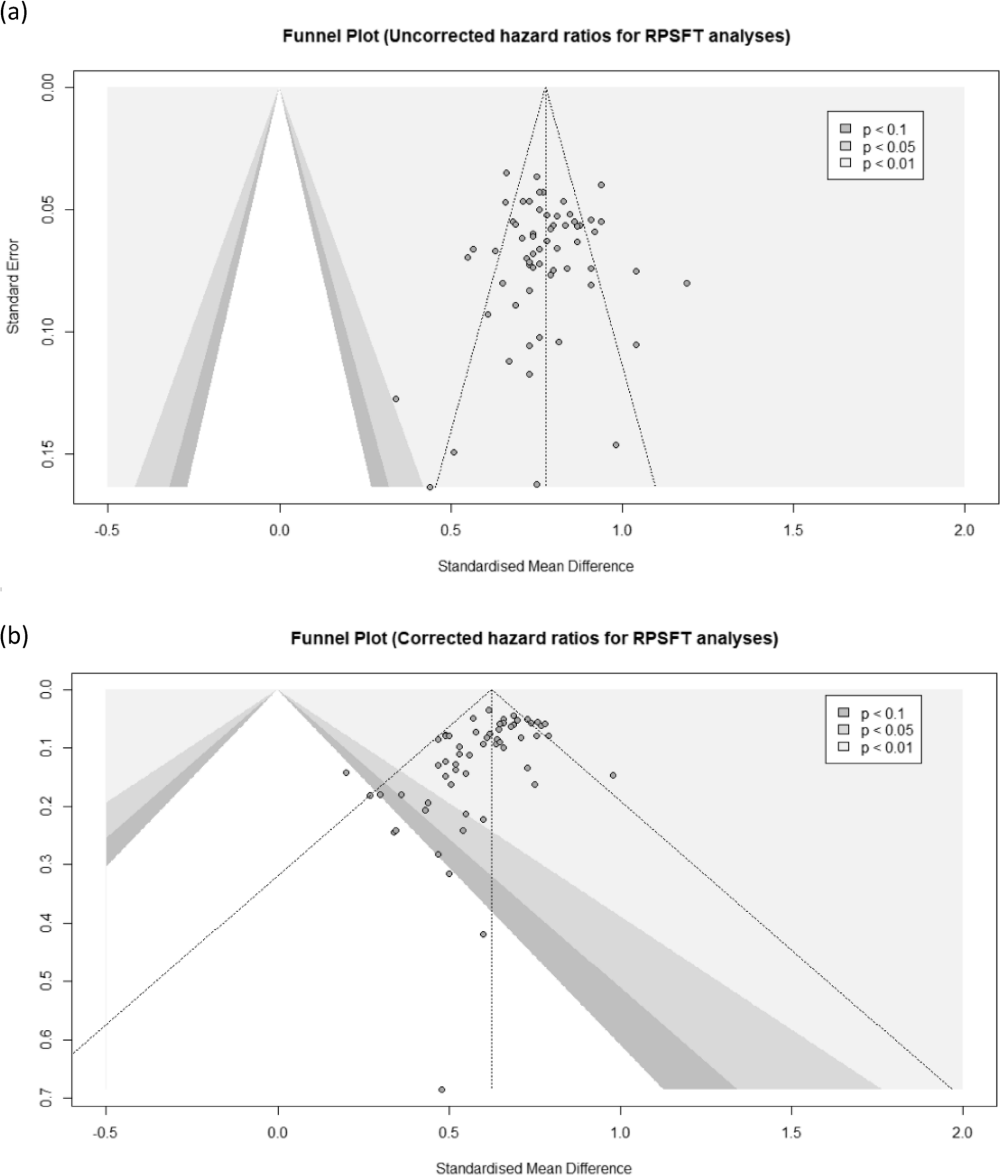
Contour-enhanced funnel plot of publication bias in **a** uncorrected and **b** corrected oncology RPSFT analyses

When using the Fleiss kappa statistic to determine correlation beyond chance alone, we found that there was moderate agreement between having a significant OS hazard ratio in the uncorrected analysis and having a significant OS hazard ratio in the corrected analysis (agreement=72%; kappa=0.43; 95% CI: 0.38 to 0.48; *p*<0.001). The ICC for the uncorrected and uncorrected hazard ratio was 0.28 (95% CI: −0.095 to 0.59; *p*=0.10). Figure 2 shows the agreement between the two hazard ratios (Bland-Altman Plot). The contour-enhanced funnel plots (Fig. 3a and b) not only show the wider variance in hazard ratios that are corrected, compared to uncorrected, but they also show publication bias in studies reporting on RPSFT analyses.

## Discussion

Ours is the first, to our knowledge, umbrella analysis of the use of RPSFT in cancer clinical trials and its implications for inferences and results. First, we found that a sizable percentage of RPSFT studies (68%) are written by medical writers and use consulting companies. Second, we found that this method lowers the overall survival hazard ratio by a median 0.1 point, which suggests a notable impact. Third, the rate of crossover only explained 19% of the variability in the change in hazard ratios. Fourth, RPSFT was used appropriately in 19% of cases (tested for fundamental efficacy without a standard of care) but inappropriately in 81% (tested for fundamental efficacy with a standard of care or in sequence). We discuss these insights.

One concerning finding from our study is that all RPSFT analyses were either funded by drug sponsors, if funding was disclosed, and/or were written by at least one author who was employed by the drug sponsor. Furthermore, a notable percentage of studies used medical writers for reporting the results of the RPSFT analyses. Industry funding, while common, can lead to notable bias, skewing results towards the publication of favorable findings for the drug company [13]. Methodological papers on RPSFT that did not have industry ties were few, [1, 4] while papers with financial industry ties were numerous [2, 14, 15].

We found that the use of the RPSFT method lowers the overall survival hazard ratio by a median 0.1 point. This is a notable impact and rivals the impact of therapies themselves [16]. This can be compared to a previous analysis that reported a pooled hazard ratio to be 0.77 for all approved cancer drugs [17], and yet almost 20% of the drugs in our analysis were not approved at the time of manuscript preparation.

In our study, we found that the correlation between the uncorrected and corrected OS hazard ratio and the percentage of individuals who crossed over to the experimental drug was low, suggesting that only a small portion of an RPSFT corrected hazard ratio is due to the percentage of control arm participants who crossover at progression. Furthermore, most studies (~52%) were conducted in situations where the drug was being tested for fundamental efficacy when there was a standard of care, situations where it is often inappropriate to cross patients over to the drug being tested. And, another 29% of studies tested a drug that was already used in a latter line, being moved upfront, where some percentage of the control arm eventually received that therapy, rendering an inappropriate situation for RPSFT analysis.

We found that only about one-quarter of studies tested a drug’s sequential efficacy and another 18% tested a drug’s fundamental efficacy when there was no standard of care. Some researchers assert that crossover is an important element in randomized trials because of the ethics of providing patients who have progression with treatment options [18]. We contend that while this is true when there are no post-progression treatment options available or the tested drug is already approved in a latter line, there are other situations where crossover is not appropriate [5]. Therefore, crossover, and methods to adjust for its effects, should not be applied generally.

Researchers have justified the use of RPSFT as a way to correct for crossover, and many have insisted that because of numerically lower hazard ratios using the RPSFT adjustment, the drug likely provided OS benefit. However, we found a moderate agreement between finding a significant OS hazard ratio in the uncorrected and corrected analysis, suggesting that even with correction for crossover, the significance of OS findings is often not changed with the use of RPSFT. In other words, RPSFT correction often does not result in a significant OS hazard ratio. Furthermore, an improvement in OS benefit is likely due to a biased overestimation of a drug’s effect, which has been previously reported [19]. This bias may be due to physicians who are more likely to prescribe crossover treatment to people who are healthier and will do better regardless of subsequent treatment [14].

There have been several recent examples of an RPSFT analysis being incorporated into FDA submission data [20–22]. In these cited examples, the corrected OS data were found to be inappropriate for or were discouraged from determining drug efficacy and had or would have no bearing on the drug’s approval. But it is concerning that drug manufacturers are beginning to incorporate these data into drug approval data. We encourage regulatory agencies and reviewers of drug data to uphold standards of appropriateness in crossover and accompanying analysis.

Other classification systems have been proposed for interpreting correlation values [23, 24]. Using these interpretations, the correlations were low to moderate, depending on whether the drugs were being tested for fundamental or sequential efficacy.

### Strengths and limitations

There are at least 3 strengths and 3 limitations. The first strength is that this is the first umbrella analysis of RPSFT analyses. Second, we characterized the appropriateness of crossover and RPSFT analysis, based on whether the situation tested a drug’s fundamental efficacy without a standard of care, tested a drug’s fundamental efficacy with a standard of care, or tested the drug in sequence, which has previously not been done. Our methods have identified limitations of RPSFT use. Third, we determined who funded and wrote the publications of the RPSFT analyses, thus identifying the sources of RPSFT analyses.

One limitation to our analysis is that our search may not have been exhaustive and did not include all studies with an RPSFT correction. Our study search was systematic and included multiple search engines, and our results should not have been differentially affected. Second, we included abstracts that had limited data reported in them. For studies that were missing key data points, we searched clinicaltrials.gov for other publications that might contain pertinent information. Finally, our findings are likely not generalizable to oncology at-large, because all the studies in our analysis were funded by the drug sponsor who has a financial interest in only publishing favorable results for their drug.

## Conclusion

In conclusion, RPSFT is a common tactic used to reinterpret trial results. The majority of this use is by the industry or through medical writers. The tactic lowers the OS hazard ratio by a median of 0.1. Only 18% of the reduction in hazard ratio is explained by rate of crossover. Nineteen percent of the time RPSFT use is appropriate, but its use is inappropriate in 81% of instances. In 29% of instances, a drug is already used as standard of care (salvage) but being tested in an earlier line. In these situations, crossover should be encouraged since it is standard of care, and RPSFT adjustment would be inappropriate. We recognize that while crossover can bias OS results, the allowance of crossover in a clinical trial and the handling of crossover in the analysis should be limited to appropriate circumstances.

## Supplementary Information

**Additional file 1: Supplemental Figure 1.** Linear regression diagnostic plots for oncology studies reporting on rank preserving structural failure time. A) All studies. B) With one outlier removed. **Supplemental Figure 2.** Identification of Rank preserving structural failure time analyses in oncology trials.

## Data Availability

Not applicable.

## Abbreviations

FDA: Food and Drug Administration
OS: Overall survival
RPSFT: Rank preserving structural failure time
SOC: Standard of care
