# Supplementary material for: Cross-sectional analysis characterizing the use of rank preserving structural failure time in oncology studies: changes to hazard ratio and frequency of inappropriate use
Source: Trials. 2023 Jun 3;24:373. doi: 10.1186/s13063-023-07412-y (PMC10239129; doi:10.1186/s13063-023-07412-y)

Supplemental Figure 1. Linear regression diagnostic plots for oncology studies reporting on rank preserving structural failure time. A) All studies. B) With one outlier removed.

A)


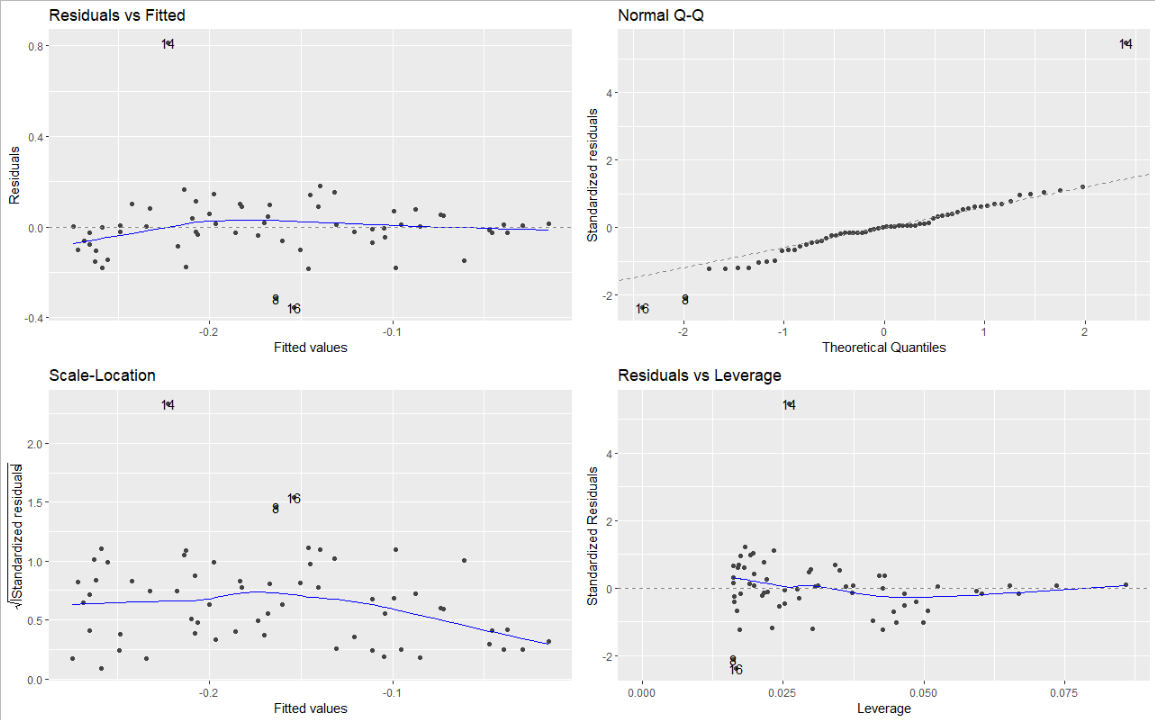


B)


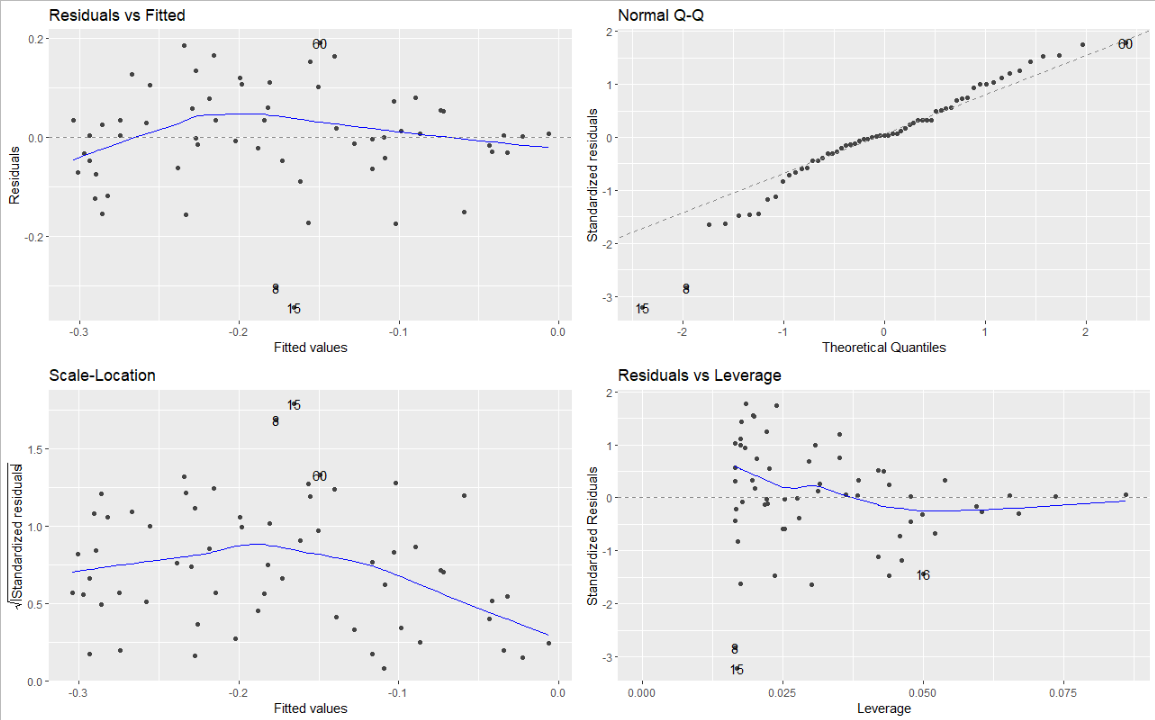

Supplement: Supplementary file 1 — Additional file 1: Supplemental Figure 1. Linear regression diagnostic plots for oncology studies reporting on rank preserving structural failure time. A) All studies. B) With one outlier removed. Supplemental Figure 2. Identification of Rank preserving structural failure time analyses in oncology trials. [file 13063_2023_7412_MOESM1_ESM.docx]
